# Chemotherapy-Induced Toxicities and Their Associations with Clinical and Non-Clinical Factors among Breast Cancer Patients in Vietnam

**DOI:** 10.3390/curroncol29110653

**Published:** 2022-10-31

**Authors:** Sang M. Nguyen, Anh T. Pham, Lan M. Nguyen, Hui Cai, Thuan V. Tran, Xiao-Ou Shu, Huong T. T. Tran

**Affiliations:** 1 Division of Epidemiology, Department of Medicine, Vanderbilt Epidemiology Center, Vanderbilt-Ingram Cancer Center, Vanderbilt University School of Medicine, Nashville, TN 37203, USA; 2 Vietnam National Cancer Institute, National Cancer Hospital, Hanoi 10000, Vietnam; 3 Hanoi Medical University, Hanoi 10000, Vietnam; 4 Hanoi Oncology Hospital, Hanoi 10000, Vietnam; 5 Ministry of Health, Hanoi 10000, Vietnam

**Keywords:** breast cancer, chemotherapy-induced toxicity, Vietnam

## Abstract

Understanding the burden and factors related to chemotherapy-induced toxicity is important in treatment planning for breast cancer patients. We conducted a prospective study among 396 newly diagnosed and chemotherapy-treated breast cancer patients recruited in two major cancer hospitals in northern Vietnam. Toxicities were captured through medical chart reviews and patient self-reports and graded using NCI CTCAE classification. Associations for sociodemographic and clinical factors with chemotherapy-induced toxicities during first-line chemotherapy were evaluated via multivariable logistic regression. Severe (i.e., grade ≥ 3) hematological (38.6%), and gastrointestinal (12.9%) toxicities were common. A pre-existing nephrological condition was significantly associated with the risk of severe hematological toxicity with adjusted odds ratios (OR) and 95% confidence intervals (CIs) of 2.30 (1.32–4.01). Patients living in rural areas had a lower risk of severe hematological toxicity (OR = 0.48; 95% CI, 0.30–0.77). Patients diagnosed with stage II and stage III–IV had a lower risk of severe gastrointestinal toxicity with ORs and 95% CIs of 0.26 (0.12–0.59) and 0.47 (0.20–1.10), respectively. Triple-negative/basal-like subtype was associated with a higher risk of severe hematological (OR = 3.15; 95% CI, 1.56–6.34) and gastrointestinal toxicities (OR = 3.60; 95% CI, 1.45–8.95) comparing to hormone receptor (HR)-positive HER2-negative subtype. Further research investigating underlying mechanisms would facilitate the development and delivery of personalized treatment and care plans.

## 1. Introduction

Adjuvant systemic treatments, before or following primary breast cancer treatment by surgery with or without radiation, have contributed to reducing cause-specific mortality from breast cancer for decades [1,2,3]. Although endocrine therapy and targeted therapy have made noteworthy progress, chemotherapy continues to have a dominant role in the clinical treatment of breast cancer. Many randomized trials have demonstrated that adjuvant chemotherapy can prevent recurrence and prolong survival [4]. However, chemotherapy can lead to varied acute side effects and long-term toxicities, affecting treatment compliance, efficacy, and long-term outcomes [5]. Although recent advances in chemotherapy have achieved more tolerable and safer outcomes, up to 87% of people experienced at least one side effect/adverse event during and after treatment [6,7]. The most common acute toxicities associated with almost all chemotherapeutic agents include myelosuppression (e.g., neutropenia nadir, anemia, leukopenia, thrombocytopenia), febrile neutropenia, alopecia, and gastrointestinal (GI) toxicities such as nausea, vomiting, diarrhea, stomatitis, and constipation. These hematological and GI toxicities are seen in 40% to 80% of breast cancer patients receiving neoadjuvant or adjuvant chemotherapy [4,8,9]. If persistent, chemotherapy-induced toxicities may adversely affect breast cancer patients’ physical health, quality of life, and emotional state. Severe chemotherapy-induced toxicities may put cancer patients at risk of dose delay or dose reduction, treatment discontinuation [10], and costly healthcare service use [11]; some may result in premature death [12,13,14]. Much evidence has shown that patients who receive a low dose of chemotherapy have reduced survival rates [15].

Almost all reports of chemotherapy-induced toxicities and their frequency came from clinical trials of new treatments and used clinician-reported toxicity ratings [7,16,17,18]. It is well documented that self-reported and clinically assessed toxicity differs [19,20] and also that clinical trial participants tend to be healthier patients. Results from clinical trials may not reflect the frequency, severity, and burden of chemotherapy-induced toxicities in breast cancer patients who receive real-world care. Currently, there are very few observational studies on chemotherapy-induced toxicity in breast cancer patients at a time when chemotherapy includes anthracycline-, taxane-, and non-anthracycline-based regimens, particularly in low-and -middle-income countries such as Vietnam.

Breast cancer is the most common cancer and the fourth leading cause of cancer-related death among Vietnamese women [21]. Systemic chemotherapy is the mainstay of breast cancer treatment in Vietnam. A guideline for breast cancer treatment was released in July 2018, which is partially adopted from the National Comprehensive Cancer Network (NCCN) guidelines and European societies such as the European Society for Medical Oncology (ESO-ESMO) international consensus guidelines [22]. A dose-dense doxorubicin and cyclophosphamide (AC) with sequential paclitaxel, a dose-dense AC followed by sequential weekly paclitaxel, and docetaxel plus cyclophosphamide (TC) are preferred and recommended to be administered with myeloid growth factor support [22]. To our knowledge, no study has been conducted among breast cancer patients to investigate chemotherapy-induced toxicity systematically in Vietnam. Population-specific information on the burden and factors related to chemotherapy-induced toxicity, which is critically important for treatment planning and decision-making, is lacking.

To address the unmet clinical need, we analyzed data from 396 breast cancer patients who were enrolled in the Vietnam Breast Cancer Study (VBCS) and received systematic chemotherapy. Using this data, we investigated the occurrence of chemotherapy-induced toxicities and evaluated their associations with patients’ demographics and clinical features among Vietnamese women with breast cancer.

## 2. Materials and Methods

### 2.1. Study Population

Our study was based on a prospective follow-up of 501 newly diagnosed Vietnamese breast cancer patients recruited into the VBCS, a case–control study of breast cancer. Details of designs and methods for the VBCS have been described [23,24]. Briefly, patients were recruited from July 2017 through June 2018 from inpatient surgical units and chemotherapy inpatient and outpatient units of two major cancer hospitals in North Vietnam: the Vietnam National Cancer Hospital and Hanoi Oncology Hospital (response rate of 93.1%). Study eligibility criteria included patients who were newly diagnosed with primary breast cancer, were 18 to 79 years old, had no prior chemotherapy, and were able to provide both verbal and written informed consent. We excluded women with a history of other cancers or concurrent life-threatening illnesses (e.g., stroke, heart failure) from the study. Written consent was obtained from all VBCS participants. Approval for human subject research was obtained from the Vietnam National Cancer Institute and Vanderbilt University Medical Center [23,24].

In-person interviews were administered at enrollment by trained interviewers using a structured questionnaire. Information collected included demographics, socioeconomic characteristics, and lifestyle factors. Participant follow-up was conducted via interviewer-administered surveys for a current health condition (i.e., quality of life and self-reported chemotherapy side effects), cancer recurrence, and vital status after study enrollment by using telephone calls or through social networks at the first follow-up (~6–11 months) and the second follow-up (~12–18 months). Clinical information was collected by reviewing patients’ medical records. We used Research Electronic Data Capture (REDCap) to manage the survey and clinical data [25].

For the current study, we excluded participants who were subsequently confirmed to have a benign tumor based on pathological reviews (*n* = 9), those diagnosed at stage 0 (*n* = 2), and participants with incomplete medical chart reviews or missing treatment information (*n* = 42). We further excluded patients who did not receive adjuvant chemotherapy (*n* = 62) and those who received concurrent radiotherapy (*n* = 11) at the first-line treatment. Finally, 396 breast cancer cases were included in the analysis (Figure S1).

### 2.2. Chemotherapy-Induced Toxicity Assessment

Breast cancer patients routinely have blood and urine tests before each cycle of chemotherapy or hospital visit to assess their health condition and chemotherapy-induced side effects. This is to assist physicians’ decisions on prescribing a treatment regimen and dosing. All test results are included in the medical records. Trained study staff reviewed medical charts and abstracted information on test dates, hemoglobin (Hgb), white blood cells (WBC), absolute neutrophil count (ANC), lymphocytes, platelets (PLT), total bilirubin, serum glutamic-oxaloacetic transaminase (SGOT), serum glutamic-pyruvic transaminase (SGPT), creatinine, proteinuria, and hematuria, and directly entered this information into the REDCap data management platform. We conducted a medical chart review approximately 18 months after completion of the study recruitment, and the time covered by medical chart review is the start of first-line chemotherapy until 31 May 2020. Medical chart reviews were completed for 99% of participants who received chemotherapy. Chemotherapy-induced toxicities, including neutropenia, anemia, lymphopenia, thrombocytopenia, hyperbilirubinemia, high SGOT or SGPT, evaluated creatinine, proteinuria, and hematuria were then graded according to the National Cancer Institute Common Toxicity Criteria of Adverse Events (NCI CTCAE) classification version 2.0. The study outcomes are the highest grade of toxicities experienced during first-line chemotherapy treatment until the first day of radiotherapy for patients who received chemotherapy and sequential radiotherapy and during first-line chemotherapy treatment through 90 days after the treatment for patients who received only chemotherapy without radiotherapy. In terms of the sequential anthracycline and taxane regimen at first-line treatment, anthracycline-induced and taxane-induced toxicity grades and dates that reached the highest grade of toxicities were captured (Figure S2). Combined hematological toxicity refers to having any of the four toxicities: neutropenia, anemia, lymphopenia, or thrombocytopenia. Combined nephrotoxicity includes evaluated creatinine, proteinuria, or hematuria, whereas combined hepatotoxicity was identified as high levels of bilirubin, SGOT, or SGPT.

GI toxicities were identified through a combination of patient self-reported side effects at the two follow-ups and the assessments recorded by treating physicians/nurses during each cycle of chemotherapy/hospital visit. Patients’ self-reported side effects on non-hematological toxicities, such as GI toxicities, have demonstrated validity and reliability [19,20]. In terms of patient self-reported side effects, participants were asked in face-to-face or telephone interviews at the two follow-ups if they had experienced nausea, vomiting, diarrhea, constipation, sore mouth, or pain or difficulty swallowing after receiving chemotherapy. These symptoms might also be documented in medical records, but details vary. Moderate and severe symptoms that required clinical intervention were more likely to be documented at each cycle of chemotherapy/hospital visit. We combined self-reported symptoms and medical chart information and graded these toxicities. The moderate and severe levels of side effects in the patient self-report form were considered as grade 2 and 3 or above NCI CTCAE classification version 2.0 and took the highest reported/recorded toxicity as the study outcome. Combined gastrointestinal toxicity incorporated five symptoms: nausea, vomiting, diarrhea, constipation, or stomatitis. Other chemotherapy-induced toxicities, particularly acute cardiotoxicity (ischemia) and infection, were abstracted and graded based on information collected from medical charts. In addition, participants were asked if they had experienced high fever, allergic reaction, itching or rash, cough, myalgia or arthralgia (muscle or joint pain), peripheral neuropathy (tingling or numbness in hands), or fatigue (feeling weak) at the two follow-ups.

In the current study, we focused on two major acute toxicities, including combined hematological toxicity and combined gastrointestinal toxicity. Since grade 3 to 4 toxicities (according to NCI CTCAE classification) might lead to changes in a patient’s management (e.g., treatment delays, dose reductions, or chemotherapy discontinuance), toxicities were grouped as dichotomous variables (i.e., grade ≥ 3 vs. grade < 3) for evaluating the associations with sociodemographic and clinical factors. Due to the rarity of severe (grade ≥ 3) hepatotoxicity and nephrotoxicity (2.0% and 0.5%) and the incidence of cardiotoxicity (no reported ischemia cases), these types of toxicities were not included in this study. In addition, we did not evaluate febrile neutropenia in this study because of the lack of a reliable assessment. The rate of documented infection and self-reported moderate/severe fever was 1.8% and 3.0%, respectively, in our study population. Finally, because we considered neuropathy as long-term toxicity, we did not include this toxicity in our analysis, although many participants self-reported moderate/severe tingling or numbness in their hands (16.7%).

### 2.3. Statistical Analyses

We described the frequency and severity of combined hematological toxicity, combined GI toxicity, and specific toxicities in the overall analyses and by chemotherapy regimens. We also described the frequency and severity of hematological and GI toxicities by selected demographic characteristics and clinical factors. The differences across subgroups were compared using chi-square tests for categorical variables. Associations of sociodemographic and clinical factors with chemotherapy-induced toxicities, including combined hematological toxicity and combined GI toxicity, were evaluated by multivariable logistic regression analysis. Adjusted odds ratios (OR) and 95% CIs were derived from logistic regression models. Potential confounders adjusted in multivariable model 1 include age groups at diagnosis (<40, 40–49, 50–59, and 60+), income level (tertile distribution), and residence (urban/rural). Multivariable model 2 added additional adjustment for BMI levels (underweight, normal weight, overweight, and obese), comorbidity (yes/no), pre-existing hematological, nephrological, and hepatological conditions (yes/no), TNM cancer stage (stage I, stage II, and stage III–IV), breast cancer subtype (HR+/HER2-negative, HR+/HER2-positive, HER2 enriched, and triple-negative//basal-like), sequential anthracycline and taxane (yes/no), and dose-dense chemotherapy (yes/no). Using granulocyte colony-stimulating factor (G-CSF), drug dose reduction and chemotherapy discontinuance were not included in the multivariable models because they all occurred after the appearance of chemotherapy-induced toxicities. All statistical analyses were performed at a 2-sided significance level of 0.05 using Stata 14.0 software package (StataCorp LLC, College station, Texas, DC, USA).

## 3. Results

Over half (54.8%) of 396 participants were diagnosed at stage II, while 21.2% and 4.8% were diagnosed at stage III and IV. Tumor stages T2, N0, and M0 were the most frequent among breast cancer patients. The percentage of breast cancer patients with ER+, PR+, and HER2-positive was 61.6%, 56.1%, and 46.2%, respectively. Most participants had HR+/HER2-negative subtypes (41.2%). The percentage of breast cancer patients with HER2 enriched and triple-negative/basal-like subtypes was 21.7% and 12.6%, respectively (Table S1). Moreover, almost all patients (94.4%) had breast cancer surgery, and most (84.1%) received adjuvant chemotherapy. Chemotherapy with sequential anthracycline and taxane was the most common (70.7%) chemotherapy regimen, with paclitaxel being the predominant taxane used, such as AC-P/FAC-P/EC-P/FEC-P. Paclitaxel was more commonly used than docetaxel (63.6% vs. 25.0%). In addition, approximately 21.0% of patients were also treated with Fluorouracil (5FU), frequently used in combination with anthracycline. Around 12% of participants were treated with dose-dense chemotherapy, and 26% received G-CSF during chemotherapy. In our study, relative dose intensity [RDI] <85% was considered a proxy for drug dose reduction, and 17.9% of patients had a dose reduction. Last but not least, 33 patients (8.3%) had chemotherapy discontinuance at the first-line setting (Table S2).

**Figure 1:**
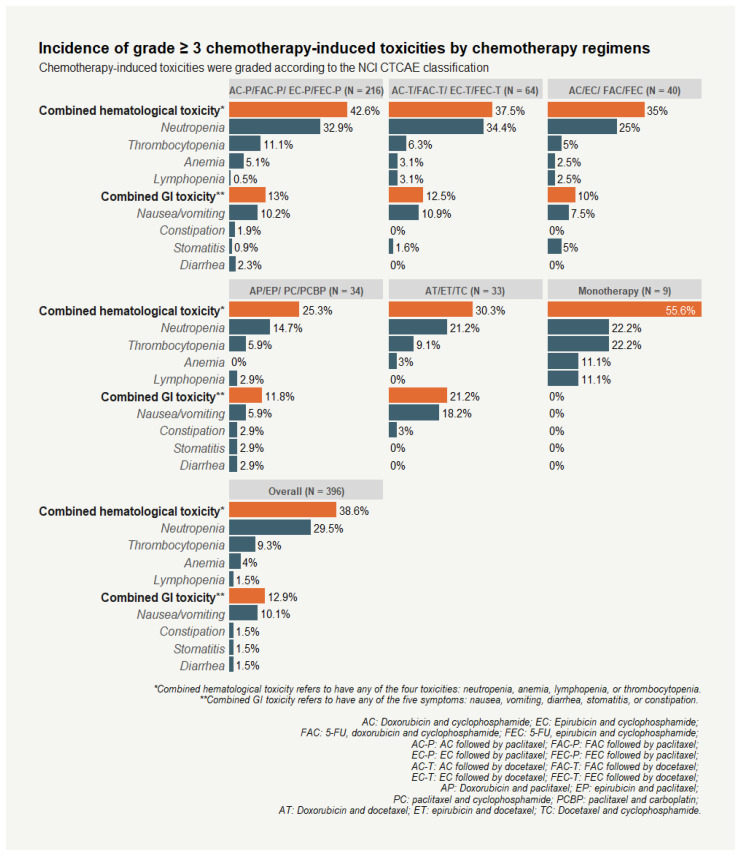
Incidence of grade ≥ 3 chemotherapy-induced toxicities among breast cancer patients by chemotherapy regimens.

Combined hematological and GI toxicities were prevalent during first-line chemotherapy treatment, with 38.6% and 12.9% of breast cancer patients experiencing grade 3 and above (grade ≥ 3) combined hematological and GI toxicity, respectively. Neutropenia was the most common chemotherapy-induced toxicity among hematological toxicities, with 29.5% of patients experiencing grade ≥ 3 toxicity. In addition, the incidence rate of grade ≥ 3 nausea/vomiting was 10.1%, making this toxicity the most frequent GI toxicity among breast cancer patients (Figure 1).

Our study population was more likely to receive sequential anthracycline and taxane than other regimens (70.3% vs. 29.3%). Significantly higher incidences of grade ≥ 3 combined hematological toxicity (41.4%), and grade ≥ 3 combined GI toxicity (12.9%) were experienced by patients receiving sequential anthracycline and taxane. Grade ≥ 3 neutropenia was also more frequently recorded among patients receiving sequential anthracycline and taxane than other regimens (33.2% vs. 20.7%; *p* = 0.02). No significant differences were observed among participants receiving sequential anthracycline and taxane for all hematological toxicities when the timing of toxicity was considered (i.e., toxicity induced by anthracycline or by taxane post anthracycline). However, the incidence and severity of GI toxicities decreased after breast cancer patients began taxane treatment (Table 1). Compared with grade ≥ 3 taxane-induced GI toxicities, patients experienced more grade ≥ 3 anthracycline-induced nausea/vomiting (7.1% vs. 2.5%). The incidences of anthracycline-induced and taxane-induced toxicities did not vary by drug combination (e.g., concurrently used with or without 5-FU [AC/EC vs. FAC/FEC]) or taxane types (paclitaxel vs. docetaxel).

Table 2 shows the incidence of combined hematological toxicity (grade ≥ 3 vs. grade < 3) and combined GI toxicity (grade ≥ 3 vs. grade < 3) by selected patients’ demographic characteristics and clinical features. A significantly higher incidence of grade ≥ 3 combined hematological toxicity was experienced by patients who used G-CSF, had drug dose reduction, or had chemotherapy discontinuance. In addition, grade ≥ 3 combined hematological toxicity was significantly more frequent among patients with triple-negative/basal-like and HR+/HER2-positive subtypes than other remaining subtypes. Patients living in rural areas and with comorbidities had lower grade ≥ 3 combined hematological toxicity than patients living in urban areas and those who had no comorbidities. GI toxicity occurrence did not vary by demographic characteristics or clinical factors, with the exception of TNM cancer stage and chemotherapy discontinuance. A significantly higher incidence of grade ≥ 3 combined GI toxicity was observed among stage I breast cancer patients and patients who discontinued their chemotherapy.

Multivariable analyses showed breast cancer patients with pre-existing nephrological conditions had a significantly higher risk of grade ≥ 3 combined hematological toxicity (OR = 2.30; 95% CI: 1.32–4.01), while patients with comorbidities had a significantly lower risk of ≥3 combined hematological toxicity (OR = 0.49; 95% CI: 0.24–0.97). In addition, patients with HR+/HER2-positive (OR = 1.78, 95% CI: 1.02–3.10) and triple-negative/basal-like (OR = 3.15, 95% CI:1.56–6.34) subtypes were more likely to experience grade ≥ 3 combined hematological toxicity than patients with HR+/HER2-negative subtype (Table 3).

Despite patients who received chemotherapy with sequential anthracycline and taxane or dose-dense chemotherapy being more likely to experience higher grade ≥ 3 combined hematological toxicity, we found no significant association in multivariate analyses (Table 3). Conversely, sequential anthracycline and taxane was not associated with an increased risk of grade ≥ 3 neutropenia (OR = 1.74; 95% CI: 0.96–3.17), but patients who received dose-dense chemotherapy were more likely to experience grade ≥ 3 neutropenia (OR = 2.64; 95% CI: 1.32–5.25). A significantly higher risk of grade ≥ 3 neutropenia was found for those with a pre-existing nephrological condition (OR = 1.86; 95%: 1.04–3.30) but not for those with comorbidities (OR = 0.56; 95% CI: 0.26–1.17). In addition, only breast cancer patients who had triple-negative/basal-like subtypes were more likely to experience grade ≥ 3 neutropenia (OR = 2.53; 95% CI: 1.22–5.24) compared to patients with HR+/HER2-negative subtype (Table S3). Breast cancer patients who were living in rural areas were less likely to experience grade ≥ 3 combined hematological toxicity (OR = 0.48; 95% CI: 0.30–0.77) and grade ≥ 3 combined neutropenia (OR = 0.53; 95% CI: 0.32–0.87) compared with those living in urban areas (Table 3 and Table S3).

Multivariable analyses showed that patients diagnosed at stage II and stage III–IV had a significantly lower risk of grade ≥ 3 combined GI toxicities than patients diagnosed at stage I (Table 4). Adjusted ORs and 95% CIs for grade ≥ 3 combined GI toxicity were 0.26 (0.12–0.59) and 0.47 (0.20–1.10) for cancer stage II and cancer stage III–IV. A similar association pattern was observed for grade ≥ 3 nausea/vomiting and TNM cancer stages. Adjusted ORs and 95% CIs for grade ≥ 3 nausea/vomiting were 0.17 (0.07–0.41) and 0.29 (0.11–0.76) for cancer stage II and cancer stage III–IV (Table S4). Patients with the triple-negative/basal-like subtype were more likely to experience grade ≥ 3 combined GI toxicity (OR = 3.60; 95% CI: 1.45–8.95) than patients with HR+/HER2-negative subtype. However, the significantly positive association with triple-negative/basal-like subtype was not observed for grade ≥ 3 nausea/vomiting (Table 4 and Table S4).

## 4. Discussion

In this study of 396 Vietnamese breast cancer patients who received chemotherapy as their first-line treatment, we found that a substantial proportion of them experienced severe (grade ≥ 3) hematological (38.6%) and GI (12.9%) toxicities. Patients who received sequential anthracycline and taxane treatment were more likely to experience severe chemotherapy-induced hematological and GI toxicities than other regimens. In multivariable analyses, patients living in rural areas showed a lower risk of severe combined hematological toxicity than those in urban areas. In addition, we found that a pre-existing nephrological condition was significantly associated with an increased risk of severe combined hematological toxicity and neutropenia. We also found a significantly positive association between severe neutropenia and dose-dense chemotherapy. Furthermore, a significantly lower risk of severe combined GI toxicity overall, and nausea/vomiting in the toxicity-specific analysis, was observed for patients diagnosed at stage II and stage III–IV. Finally, triple-negative/basal-like breast cancer was significantly associated with high risks of severe chemotherapy-induced hematological and GI toxicities compared with HR+/HER2-negative subtypes.

The incidence and severity of reported toxicities varied widely in previous studies due to various study designs, study populations, and sources of outcome data (i.e., clinician assessment and patient reports) [19,20]. However, our results are generally consistent with a recent systematic review and meta-analysis of seven clinical trials [26]. Both hematological and GI toxicities, such as neutropenia, nausea, vomiting, and mucositis, were common with breast cancer chemotherapy and were largely more severe in anthracycline-based regimens [26]. Moreover, among participants receiving sequential anthracycline and taxane, the incidence and severity of GI toxicities decreased after breast cancer patients began taxane. No significant differences were observed for hematological toxicities, whether they occurred during anthracycline treatment or during taxane administration post anthracycline treatment. Our findings are in line with the previous studies and support the evidence that adding taxane (e.g., paclitaxel) sequentially to the anthracycline-based regimens does not increase the overall incidence and severity of toxicity [27,28].

Few observational studies have investigated chemotherapy-induced adverse effects and factors associated with the severity of toxicities in breast cancer chemotherapy, including anthracycline-, taxane-, and non-anthracycline-based regimens. A 2014 population-based study evaluated the rates of first hospitalization (i.e., interpreted as severe levels) for eight reasons, including neutropenia, infection, fever, thrombocytopenia, anemia, dehydration, delirium, and other adverse effects of chemotherapy that occurred within six months of chemotherapy initiation among 3567 breast cancer patients older than 65 from the SEER/Texas Cancer Registry-Medicare database, and 9327 patients younger than 65 from the MarketScan database, diagnosed with stages I–IV breast cancer from 2003–2007 [29]. The study reported that among patients younger than 65, the unplanned hospitalization rates ranged from 6.2% (dose-dense AC followed by paclitaxel) to 10.0% (TAC: docetaxel, doxorubicin, plus cyclophosphamide every three weeks); and around 6% of patients were hospitalized for neutropenia, fever, or infection. Among patients older than 65, the rate of those admitted to the hospital ranged from 12.7% (TC: docetaxel and cyclophosphamide every three weeks) to 24.2% (TAC); and 12.4% of patients were hospitalized for neutropenia, fever, or infection [29]. In addition, the study suggested the regimens TAC and AC followed by docetaxel every three weeks (AC-T) were associated with the highest risk of chemotherapy-related hospitalization compared with the TC regimen for all age groups [29]. Additionally, the benefits of prophylactic G-CSF in reducing chemotherapy-related hospitalization rates and improving the ability of elderly patients to complete all cycles of chemotherapy were seen in SEER-Medicare patients [30,31,32]. Studies have shown that Asians may experience a higher incidence and severity rate of hematological toxicity than Caucasians even with G-CSF use [33]. Specifically, breast cancer patients in Asia-Pacific regions receiving docetaxel-containing regimens (e.g., the regimens of TAC or TC) or dose-dense chemotherapy (dose-dense AC followed by docetaxel) had a high incidence of neutropenia (20–41%) [34,35].

In our study, few patients received prophylactic G-CSF treatment, likely due to limited resources, which may explain why a substantial proportion of breast cancer patients experienced severe (grade ≥ 3) hematological toxicity. In Vietnam, the National Health Insurance has approved reimbursement for myeloid growth factor support only when the presence of chemotherapy-induced toxicities is documented. Therefore, G-CSF was often offered after the appearance of chemotherapy-induced toxicities among Vietnamese breast cancer patients, as observed in our study (i.e., patients who received G-CSF were more likely to be those with severe hematological toxicities). In addition, our study found that patients who received dose-dense chemotherapy were more likely to have severe neutropenia. Over half (52.1%) of patients who had drug dose reduction and most patients (97.0%) who had chemotherapy discontinuance experienced severe combined hematological toxicity. These results highlight the importance and necessity of prophylactic G-CSF for breast cancer chemotherapy in Vietnam.

A study based on SEER registries evaluated 1945 women aged 20–79 diagnosed with early-stage breast cancer from 2013 to 2014 for seven toxicities, including nausea/vomiting, diarrhea, constipation, pain, arm edema, dyspnea, and breast skin irritation [19]. Approximately 45% of patients reported at least one severe/very severe toxicity using a patient-reported toxicity rating on a 5-point Likert scale, 9% reported unscheduled clinic visits for toxicity management, and 5% visited an emergency department or hospital approximately seven months after diagnosis. Nearly 25% of chemotherapy recipients reported severe/very severe nausea/vomiting during their cancer treatment [19]. However, GI toxicities were less common in our study participants. Our two follow-up surveys were carried out from 6 to 11 months after study enrollment for the first follow-up and from 12 to 18 months for the second follow-up. Not assessing side effects during active chemotherapy cycles may have led to an underestimation of GI toxicities because most were based on self-reports. However, severe GI toxicities are less likely to be missed since, as our study shows, these toxicities are more likely to be associated with dose reduction and early termination of chemotherapy.

Severe chemotherapy-induced toxicities may lead to dose delay or dose reduction, chemotherapy discontinuance, and high-cost healthcare service to manage these side effects; some may result in premature death [10,11,12,13,14]. As a result, identifying patients at higher risk before chemotherapy using factors such as demographic characteristics and clinical facets may have a significant clinical impact. It may enable caregivers to initiate supportive measures before the onset of complications [36]. Previous studies have suggested that race, age, comorbidity, and BMI may be associated with chemotherapy toxicities [37,38]. Racial differences in acute toxicities were notably documented in women with breast cancer who received FEC 100 chemotherapy and TC regimen [33,39]. There was a general trend toward a higher incidence and severity of hematological toxicity experienced by Asians than Caucasians (over 30% vs. <5%) when G-CSF use was consistent. Conversely, reporting of non-hematological toxicities (~20%) did not reveal significant differences across populations for both regimens [33,39]. Being Asian and having a low BMI (underweight or normal weight, BMI < 25 kg/m^2^) were significantly associated with severe hematological toxicity during FEC 100 chemotherapy [40]. Evidence of a strong relationship between low/normal BMI and increased incidence of severe neutropenia was reported in a cohort study of 6248 women with early-stage breast cancer [9]. However, our study did not find severe hematological toxicity significantly associated with age, comorbidity, TNM cancer stage, or other evaluated factors. In our study, we found lower incidences of severe combined hematological toxicity and GI toxicity in breast cancer patients who were underweight (BMI < 18.5 kg/m^2^) or obese (≥25 kg/m^2^) compared to patients with normal weight (BMI 18.5–22.9 kg/m^2^) or who are overweight (23.0–24.9 kg/m^2^). In specific toxicity analysis, we found that overweight patients had a significantly lower risk of severe nausea/vomiting. The reasons behind the observed reduced risk of chemotherapy-induced toxicities among lean and obese cancer patients are not completely understood. One possible explanation is that obese patients are more likely to receive planned empirical dose reduction (aka “dose capping”) [41], but this cannot explain why underweight patients had low toxicities. Further investigation on pharmacokinetic profiles of chemotherapy agents among obese and underweight patients is warranted [42]. Dose capping might also partially explain the significant inverse association with severe nausea/vomiting among patients diagnosed at stage III–IV. That group had a greater representation of those who were overweight or obese in our study.

We found that a pre-existing nephrological condition (i.e., elevated creatinine, proteinuria, and hematuria before chemotherapy) was significantly associated with an increased risk of severe combined hematological toxicity and neutropenia in particular, whereas pre-existing hematological and hepatological conditions were not significantly associated with hematological toxicity. A study involving 619 patients age ≥ 65 with early-stage breast cancer who received CMF, AC, or capecitabine reported that pretreatment renal function did not influence the occurrence of hematologic toxicity regardless of regimen. Whereas an increased creatine clearance at baseline was significantly related to the high occurrence of non-hematologic toxicity for the AC regimen and very mildly for the capecitabine regimen, but not related for the CMF regimen [43]. Another study revealed a positive association between severe chemotherapy-induced toxicities and decreased creatine clearance at baseline among older patients with cancer [44]. Inconsistent with our findings, lower pretreatment blood counts (e.g., WBC, ANC) and Hgb were previously suggested to be associated with chemotherapy-induced hematological toxicities [27,36,45]. Although not confirmed, we speculate that oncologists might have considered patients’ pre-existing hematological conditions in cancer treatment. Nevertheless, our findings on renal function and chemotherapy toxicity reinforce the importance of considering renal function before administering chemotherapy.

In our study, patients living in rural areas showed a lower risk of severe hematological toxicity than those living in urban areas. The physical and financial burden on our participants who resided in rural areas, including the cost of medical care services, travel time to the hospital, and distance from patients’ homes to the hospital, might have been incorporated in clinicians’ decisions when selecting chemotherapy regimens and schedules. However, no differences in chemotherapy regimens and schedules were observed in urban and rural dwelling patients. Thus, these suggests that other factors, such as lifestyle, physical fitness/overall health status at baseline, physical activity, diet habits, gut microbiome, and family and social support, may contribute to the association between residential areas and the risk of hematological toxicity. For example, nutritional deficiencies (e.g., vitamin B12 and folate) have been associated with neutropenia [46]. Further studies are needed to understand the impact of these factors on chemotherapy-induced toxicities in Vietnam.

Evidence on the association between chemotherapy-induced toxicity and molecular subtypes of breast cancer is limited. Molecular subtypes of breast cancer have different chemotherapy regimens and schedules, therapeutic responses, and clinical outcomes [47]. Thus, chemotherapy-induced toxicities may vary by those subtypes of breast cancer. Our study found that triple-negative/basal-like breast cancer patients were significantly associated with high risks of severe chemotherapy-induced hematological and GI toxicities compared with other breast cancer subtypes, except for severe nausea/vomiting. In addition, a positive association with combined hematological toxicity was also observed for patients with HR+/HER2-positive compared with patients with HR+/HER2-negative. The more aggressive treatment experience by these patients may explain their high toxicity risks.

Our study is the first to investigate associations of severe chemotherapy-induced toxicities with demographic characteristics and clinical features among Vietnamese breast cancer patients. The availability of blood and urine test results before and during each cycle of chemotherapy/hospital visit and detailed clinical information of breast cancer patients are strengths of this study. The chemotherapy-induced hematological toxicities were captured during first-line chemotherapy treatment and 90 days after the treatment. In our research, non-hematological chemotherapy-induced toxicities (i.e., GI toxicities) were identified through a combination of patient self-reported side effects at the two follow-ups and a review of the assessments recorded by treating physicians/nurses during each cycle of chemotherapy/hospital visit. This minimized the concern related to underestimated non-hematological chemotherapy-induced toxicities by clinicians. Information on patient self-reported side effects was collected through a structured questionnaire administered by trained interviewers following a standard protocol. However, as aforementioned, our study is limited because toxicity information during active chemotherapy was not collected. In addition, the response rates for follow-up surveys were moderate for the first follow-up (77.6% after excluding deceased patients) and the second follow-up (61.9% after excluding deceased patients). Patients diagnosed at early stages were more likely to complete the first follow-up than those diagnosed at late stages. The first follow-up surveys’ response rates were 85.5%, 76.5%, and 74.8% for patients in stage I, stage II, and stage III–IV. The loss of follow-up would likely affect the statistical power for GI toxicity assessment but not hematological toxicity, as the latter was assessed solely via medical chart review. Furthermore, our study only accounted for ~35% of newly diagnosed breast cancer patients treated at Vietnam National Cancer Hospital and Hanoi Oncology Hospital from July 2017 to June 2018. Therefore, our findings may not be generalizable to all breast cancer cases, particularly those treated in other settings in Vietnam.

In conclusion, we found that a substantial proportion of breast cancer patients in Vietnam suffered severe hematological and GI toxicities during their first-line chemotherapy. Our study characterized the burden of chemotherapy-induced toxicity faced by patients, providing valuable information to assist Vietnamese oncologists/clinicians in treatment planning, dose adjustments, and management of side effects. In addition, our study calls for further research on factors related to chemotherapy-induced toxicities and factors related to interindividual variations in chemotherapy-induced toxicities that may facilitate the delivery of personalized treatment and improve treatment outcomes.

## Figures and Tables

**Table 1:** Incidence of grade ≥ 3 chemotherapy-induced toxicities by sequential anthracycline and taxane.

		Sequential Anthracycline and Taxane					
		No	Yes		Anthracycline-Induced	Taxane-Induced Post Anthracycline	
		*N* = 116	*N* = 280	*p* ^1^	*N* = 280	*N* = 280	*p* ^2^
	Grade	*n* (%)	*n* (%)		*n* (%)	*n* (%)	
**Hematological toxicity**							
Combined toxicity ^a^	≥3	37 (31.9)	116 (41.4)	0.009	79 (28.2)	65 (23.2)	0.12
Neutropenia	≥3	24 (20.7)	93 (33.2)	0.02	61 (21.8)	49 (17.5)	0.12
Thrombocytopenia	≥3	9 (7.8)	28 (10.0)	0.57	14 (5.0)	17 (6.1)	0.45
Anemia	≥3	3 (2.6)	3 (1.1)	0.36 *	1 (0.4)	2 (0.7)	0.68
Lymphopenia	≥3	3 (2.6)	13 (4.6)	0.42 *	10 (3.6)	8 (2.9)	0.45
**GI toxicity**							
Combined toxicity ^b^	≥3	15 (12.9)	36 (12.9)	0.98	25 (7.1)	10 (3.6)	0.04
Nausea/vomiting	≥3	11 (9.5)	29 (10.4)	0.79	20 (7.1)	7 (2.5)	0.04
Constipation	≥3	2 (1.7)	4 (1.4)	1.00 *	4 (1.5)	0 (0.0)	0.42
Stomatitis	≥3	3 (2.6)	3 (1.1)	0.36 *	3 (1.1)	0 (0.0)	0.03
Diarrhea	≥3	1 (0.9)	5 (1.8)	0.67 *	2 (0.7)	3 (1.1)	0.40

^1^*p*-value for chi-square tests; * *p*-value for fisher’s exact test; ^2^*p*-value for the equality of proportion; ^a^ Combined hematological toxicity refers to having any of the four toxicities: neutropenia, anemia, lymphopenia, or thrombocytopenia. ^b^ Combined GI toxicity refers to having any of the five symptoms: nausea, vomiting, diarrhea, stomatitis, or constipation.

**Table 2:** Highest grade of chemotherapy-induced toxicity by selected demographic characteristics, disease characteristics, and characteristics related to treatment among study participants (total N = 396).

		Combined GI Toxicity			Combined Hematological Toxicity		
	N	Grade < 3	Grade ≥ 3	*p* ^1^	Grade < 3	Grade ≥ 3	*p* ^1^
**Age group**							
<40	61	51 (83.6)	10 (16.4)	0.41	40 (65.6)	21 (34.4)	0.13
40–49	153	131 (85.6)	22 (14.4)		84 (54.9)	69 (45.1)	
50–59	135	119 (88.1)	16 (11.9)		85 (63.0)	50 (37.0)	
≥60	47	44 (93.6)	3 (6.4)		34 (72.3)	13 (27.7)	
**Education**							
Primary school	60	53 (88.3)	7 (11.7)	0.62	45 (75.0)	15 (25.0)	0.13
Middle school	168	150 (89.3)	18 (10.7)		98 (58.3)	70 (41.7)	
High school	98	83 (84.7)	15 (15.3)		59 (60.2)	39 (39.8)	
College or higher	70	59 (84.3)	11 (15.7)		41 (58.6)	29 (41.4)	
**Income**							
Low (T1)	141	122 (86.5)	19 (13.5)	0.53	91 (64.5)	50 (35.5)	0.62
Middle (T2)	128	109 (85.2)	19 (14.8)		77 (60.2)	51 (39.8)	
High (T3)	127	114 (89.8)	13 (10.2)		75 (59.1)	52 (40.9)	
**Residence**							
Urban area	150	131 (87.3)	19 (12.7)	0.92	80 (53.3)	70 (46.7)	0.01
Rural area	246	214 (87.0)	32 (13.0)		163 (66.3)	83 (33.7)	
**Menopausal status**							
Pre-menopausal	228	195 (85.5)	33 (14.5)	0.27	137 (60.1)	91 (39.9)	0.54
Post-menopausal	168	150 (89.3)	18 (10.7)		106 (63.1)	62 (36.9)	
**Family history of breast cancer**							
No	380	331 (87.1)	49 (12.9)	0.96	234 (61.6)	146 (38.4)	0.67
Yes	16	14 (87.5)	2 (12.5)		9 (56.3)	7 (43.8)	
**BMI levels (kg/m^2^)**							
Underweight (<18.5)	42	38 (90.5)	4 (9.5)	0.43	26 (61.9)	16 (38.1)	0.07
Normal weight (18.5–22.9)	245	208 (84.9)	37 (15.1)		153 (62.4)	92 (37.6)	
Overweight (23–24.9)	75	67 (89.3)	8 (10.7)		38 (50.7)	37 (49.3)	
Obese (≥25)	34	32 (94.1)	2 (5.9)		26 (76.5)	8 (23.5)	
**Comorbidity ^a^**							
No	330	288 (87.3)	42 (12.7)	0.84	195 (59.1)	135 (40.9)	0.04
Yes	66	57 (86.4)	9 (13.6)		48 (72.7)	18 (27.3)	
**Pre-existing hematological condition ^b^**							
No	280	240 (85.7)	40 (14.3)	0.19	173 (61.8)	107 (38.2)	0.79
Yes	116	105 (90.5)	11 (9.5)		70 (60.3)	46 (39.7)	
**Pre-existing nephrological condition ^c^**							
No	319	274 (85.9)	45 (14.1)	0.14	205 (64.3)	114 (35.7)	0.06
Yes	77	71 (92.2)	6 (7.8)		38 (49.4)	39 (50.6)	
**Pre-existing hepatological condition ^d^**							
No	330	290 (87.9)	40 (12.1)	0.31	201 (60.9)	129 (39.1)	0.67
Yes	66	55 (83.3)	11 (16.7)		42 (63.6)	24 (36.4)	
**TNM stage**							
Stage I	76	59 (77.6)	17 (22.4)	0.01	46 (60.5)	30 (39.5)	0.98
Stage II	217	197 (90.8)	20 (9.2)		134 (61.8)	83 (38.2)	
Stage III–IV	103	89 (86.4)	14 (13.6)		63 (61.2)	40 (38.8)	
**Breast cancer subtype**							
HR+/HER2-negative	163	146 (89.6)	17 (10.4)	0.09	111 (68.1)	52 (31.9)	0.01
HR+/HER2-positive	97	85 (87.6)	12 (12.4)		51 (52.6)	46 (47.4)	
HER2 enriched	86	76 (88.4)	10 (11.6)		58 (67.4)	28 (32.6)	
Triple-negative	50	38 (76.0)	12 (24.0)		23 (46.0)	27 (54.0)	
**Sequential anthracycline and taxane**							
No	116	101 (87.1)	15 (12.9)	0.98	79 (68.1)	37 (31.9)	0.01
Yes	280	244 (87.1)	36 (12.9)		164 (58.6)	116 (41.4)	
**Dose-dense chemotherapy**							
No	349	301 (86.2)	48 (13.8)	0.16	220 (63.0)	129 (37.0)	0.06
Yes	47	44 (93.6)	3 (6.4)		23 (48.9)	24 (51.1)	
**Using G-CSF**							
No	290	253 (87.2)	37 (12.8)	0.91	188 (64.8)	102 (35.2)	0.02
Yes	106	92 (86.8)	14 (13.2)		55 (51.9)	51 (48.1)	
**Drug dose reduction in chemotherapy ^e^**							
RDI ≥ 85%	325	280 (86.2)	45 (13.8)	0.22	209 (64.3)	116 (35.7)	0.01
RDI < 85%	71	65 (91.5)	6 (8.5)		34 (47.9)	37 (52.1)	
**Chemotherapy discontinuance**							
No	363	320 (88.2)	43 (11.8)	0.04	242 (66.7)	121 (33.3)	0.001
Yes	33	25 (75.8)	8 (24.2)		1 (3.0)	32 (97.0)	

^1^*p*-value for chi-square tests; ^a^ Having a diagnosis of specific comorbidities, including diabetes mellitus, hypertension, hyperlipidemia, coronary heart disease (CHD), stroke, myocardial infarction, arthritis, lupus, or another chronic disease at enrollment. ^b^ Having at least one of the hematological symptoms (grade ≥ 1), including anemia, neutropenia, lymphopenia, and thrombocytopenia within 120 days prior to chemotherapy. ^c^ Having at least one of the nephrological symptoms (grade ≥ 1), including high creatinine, proteinuria, and hematuria within 120 days prior to chemotherapy. ^d^ Having at least one of the hepatological symptoms (grade ≥ 1) including high bilirubin, SGOT, and SGPT within 120 days prior to chemotherapy. ^e^ RDI: Relative dose intensity—ratio of the dose intensity delivered to the reference standard dose intensity for a chemotherapy regimen.

**Table 3:** Association of demographic characteristics and clinical factors with combined hematological toxicity.

	Combined Hematological Toxicity (Grade ≥ 3 vs. Grade < 3)		
	No. of Grade ≥ 3/Grade < 3	Model 1	Model 2
		Adjusted OR (95% CI) ^1^	Adjusted OR (95% CI) ^2^
**Age group**			
<40	21/40	1	1
40–49	69/84	1.64 (0.88–3.07)	2.04 (1.04–4.00)
50–59	50/85	1.25 (0.66–2.39)	1.95 (0.95–4.02)
≥60	13/34	0.76 (0.33–1.76)	1.18 (0.45–3.08)
**Income levels**			
Low (T1)	50/91	1	1
Middle (T2)	51/77	1.15 (0.69–1.90)	1.15 (0.67–1.98)
High (T3)	52/75	1.12 (0.69–1.92)	1.21 (0.70–2.08)
**Residence**			
Urban area	70/80	1	1
Rural area	83/163	0.59 (0.38–0.90)	0.48 (0.30–0.77)
**BMI levels**			
Normal weight (18.5–22.9)	92/153	1	1
Underweight (<18.5)	16/26	0.99 (0.50–1.97)	0.97 (0.47–2.02)
Overweight (23–24.9)	37/38	1.61 (0.94–2.75)	1.69 (0.95–2.98)
Obese (≥25)	8/26	0.50 (0.21–1.15)	0.56 (0.23–1.36)
**Comorbidity ^a^**			
No	135/195	1	1
Yes	18/48	0.54 (0.28–1.01)	0.49 (0.24–0.97)
**Pre-existing hematological condition ^b^**			
No	107/173	1	1
Yes	46/70	1.12 (0.71–1.77)	0.90 (0.55–1.47)
**Pre-existing nephrological condition ^c^**			
No	114/205	1	1
Yes	39/38	1.90 (1.14–3.17)	2.30 (1.32–4.01)
**Pre-existing hepatological condition ^d^**			
No	129/201	1	1
Yes	24/42	0.96 (0.54–1.68)	1.11 (0.60–2.05)
**TNM stage**			
Stage I	30/46	1	1
Stage II	83/134	0.99 (0.57–1.72)	0.89 (0.48–1.62)
Stage III–IV	40/63	1.11 (0.60–2.10)	1.08 (0.55–2.12)
**Breast cancer subtype**			
HR+/HER2-negative	52/111	1	1
HR+/HER2-positive	46/51	1.85 (1.09–3.14)	1.78 (1.02–3.10)
HER2 enriched	28/58	1.08 (0.61–1.91)	0.89 (0.48–1.62)
Triple-negative	27/23	2.98 (1.52–5.83)	3.15 (1.56–6.34)
**Sequential anthracycline and taxane**			
No	37/79	1	1
Yes	116/164	1.55 (0.96–2.51)	1.47 (0.85–2.53)
**Dose-dense chemotherapy**			
No	129/220	1	1
Yes	24/23	1.86 (1.00–3.49)	1.80 (0.91–3.57)

^1^ Multivariable mode 1 was adjusted for age groups at diagnosis, income levels, and residence. ^2^ Multivariable model 2 was the multivariable model 1 with additional adjustment for BMI levels, comorbidity, pre-existing hematological, nephrological and hepatological conditions, TNM cancer stage, breast cancer subtype, sequential anthracycline and taxane and dose-dense chemotherapy. ^a^ Having a diagnosis of specific comorbidities, including diabetes mellitus, hypertension, hyperlipidemia, coronary heart disease (CHD), stroke, myocardial infarction, arthritis, lupus, or another chronic disease at enrollment. ^b^ Having at least one of the hematological symptoms (grade ≥ 1), including anemia, neutropenia, lymphopenia, and thrombocytopenia within 120 days prior to chemotherapy. ^c^ Having at least one of the nephrological symptoms (grade ≥ 1), including high creatinine, proteinuria, and hematuria within 120 days prior to chemotherapy. ^d^ Having at least one of the hepatological symptoms (grade ≥ 1) including high bilirubin, SGOT, and SGPT within 120 days prior to chemotherapy.

**Table 4:** Association of demographic characteristics and clinical factors with combined GI toxicities.

	Combined GI Toxicity (Grade ≥ 3 vs. Grade < 3)		
	No. of Grade ≥ 3/Grade < 3	Model 1	Model 2
		Adjusted OR (95% CI) ^1^	Adjusted OR (95% CI) ^2^
**Age group**			
<40	10/51	1	1
40–49	22/131	0.83 (0.36–1.88)	0.86 (0.36–2.08)
50–59	16/119	0.66 (0.28–1.57)	0.58 (0.22–1.55)
60+	3/44	0.33 (0.09–1.29)	0.20 (0.04–2.94)
**Income levels**			
Low (T1)	19/122	1	1
Middle (T2)	19/109	1.08 (0.54–2.16)	1.37 (0.64–2.95)
High (T3)	13/114	0.69 (0.32–1.49)	0.89 (0.39–2.03)
**Residence**			
Urban area	19/131	1	1
Rural area	32/214	1.02 (0.55–1.91)	1.20 (0.61–2.36)
**BMI levels**			
Normal weight (18.5–22.9)	37/208	1	1
Underweight (<18.5)	4/38	0.58 (0.19–1.74)	0.46 (0.14–1.45)
Overweight (23–24.9)	8/67	0.72 (0.32–1.64)	0.74 (0.31–1.75)
Obese (≥25)	2/32	0.36 (0.08–1.60)	0.33 (0.07–1.58)
**Comorbidity ^a^**			
No	42/288	1	1
Yes	9/57	1.53 (0.66–3.57)	1.92 (0.76–4.88)
**Pre-existing hematological condition ^b^**			
No	40/240	1	1
Yes	11/105	0.65 (0.32–1.32)	0.70 (0.32–1.51)
**Pre-existing nephrological condition ^c^**			
No	45/274	1	1
Yes	6/71	0.49 (0.20–1.20)	0.43 (0.16–1.13)
**Pre-existing hepatological condition ^d^**			
No	40/290	1	1
Yes	11/55	1.55 (0.73–3.27)	1.80 (0.80–4.06)
**TNM stage**			
Stage I	17/59	1	1
Stage II	20/197	0.32 (0.15–0.66)	0.26 (0.12–0.59)
Stage III–IV	14/89	0.51 (0.23–1.14)	0.47 (0.20–1.10)
**Breast cancer subtype**			
HR+/HER2-negative	17/146	1	1
HR+/HER2-positive	12/85	1.12 (0.51–2.49)	1.24 (0.54–2.87)
HER2 enriched	10/76	1.16 (0.50–2.71)	1.43 (0.58–3.56)
Triple-negative/basal-like	12/38	2.84 (1.23–6.56)	3.60 (1.45–8.95)
**Sequential anthracycline and taxane**			
No	15/101	1	1
Yes	36/244	0.83 (0.43–1.63)	1.29 (0.60–2.77)
**Dose-dense chemotherapy**			
No	48/301	1	1
Yes	3/44	0.43 (0.13–1.45)	0.43 (0.12–1.52)

^1^ Multivariable mode 1 was adjusted for age groups at diagnosis, income levels, and residence. ^2^ Multivariable model 2 was the multivariable model 1 with additional adjustment for BMI levels, comorbidity, pre-existing hematological, nephrological and hepatological conditions, TNM cancer stage, breast cancer subtype, sequential anthracycline and taxane and dose-dense chemotherapy. ^a^ Having a diagnosis of specific comorbidities, including diabetes mellitus, hypertension, hyperlipidemia, coronary heart disease (CHD), stroke, myocardial infarction, arthritis, lupus, or another chronic disease at enrollment. ^b^ Having at least one of the hematological symptoms (grade ≥ 1), including anemia, neutropenia, lymphopenia, and thrombocytopenia within 120 days prior to chemotherapy. ^c^ Having at least one of the nephrological symptoms (grade ≥ 1), including high creatinine, proteinuria, and hematuria within 120 days prior to chemotherapy. ^d^ Having at least one of the hepatological symptoms (grade ≥ 1) including high bilirubin, SGOT, and SGPT within 120 days prior to chemotherapy.

## Data Availability

Data is available on request. The data underlying this article will be shared upon reasonable request to the corresponding author.

## Supplementary Materials

The following supporting information can be downloaded at: https://www.mdpi.com/article/10.3390/curroncol29110653/s1, Figure S1: Flow diagram of study participant inclusion criteria; Figure S2: Timeline of chemotherapy-induced toxicity assessment; Table S1: Breast cancer patients’ sociodemographic and clinical characteristics; Table S2: First-line treatment for breast cancer among study participants; Table S3: Association of demographic characteristics and clinical factors with neutropenia; Table S4: Association of demographic characteristics and clinical factors with nausea/vomiting.
