# Supplementary material for: Chemotherapy-Induced Toxicities and Their Associations with Clinical and Non-Clinical Factors among Breast Cancer Patients in Vietnam
Source: Curr Oncol. 2022 Oct 31;29(11):8269–84. doi: 10.3390/curroncol29110653 (PMC9689154; doi:10.3390/curroncol29110653)
Supplement: Supplementary file 1 [file curroncol-29-00653-s001.zip › curroncol-1958520-supplementary.pdf]

Current Oncology

Supplementals

Chemotherapy-Induced Toxicities and Their Associations with Clinical and Non-Clinical Factors among Breast Cancer Patients in Vietnam

Sang M. Nguyen [1], Anh T. Pham [2,3], Lan M. Nguyen [4], Hui Cai [1], Thuan V. Tran [2,5], Xiao-Ou Shu [1,*] and Huong T. T. Tran [2,3,*]

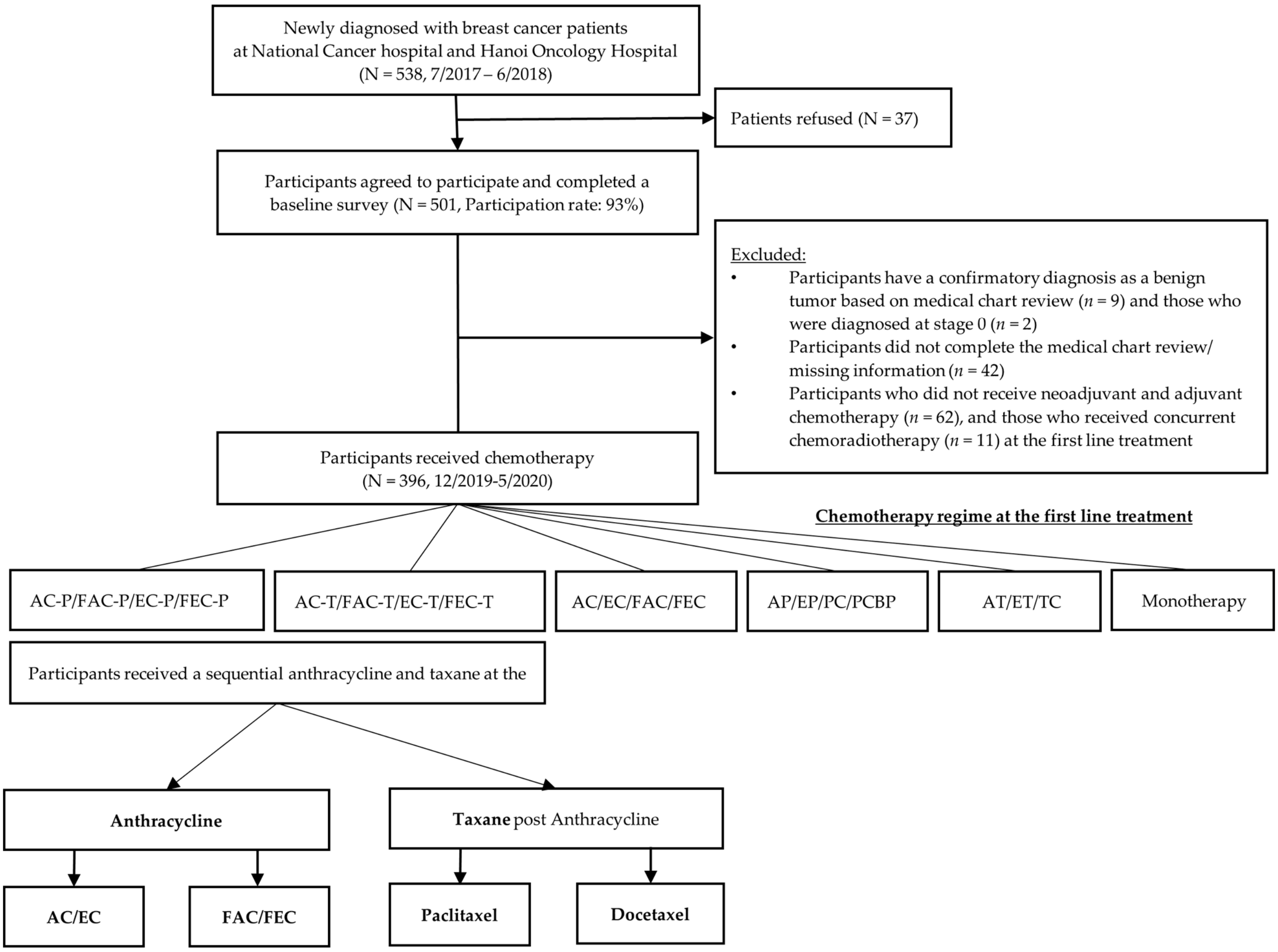

Figure S1. Flow diagram of study participant inclusion criteria.

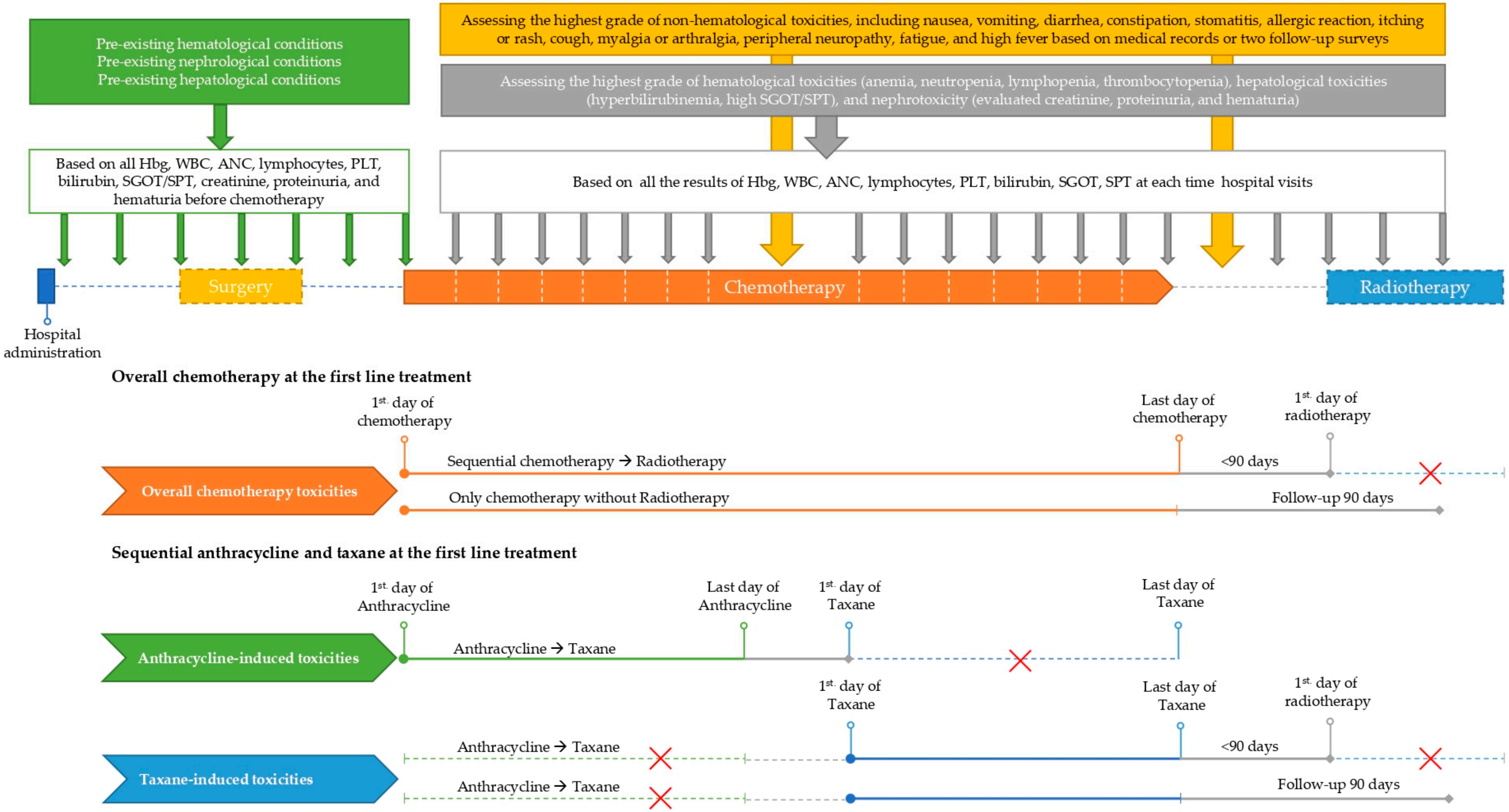

Figure S2. Timeline of chemotherapy-induced toxicity assessment.

Current Oncology

Table S1. Breast cancer patients' sociodemographic and clinical characteristics.

	All eligible participants (N = 396)			All eligible participants (N = 396)	
	n	%		n	%
**Age (Mean ± SD; years)**	49.4±9.7		**Menopausal status**		
**Age group**			Pre-menopausal	228	57.6
< 40	61	15.4	Post-menopausal	168	42.4
40-49	153	38.6	**ER status**		
50-59	135	34.1	Negative	152	38.4
≥ 60	47	11.9	Positive	244	61.6
**Education levels**			**PR status**		
Primary school	60	15.2	Negative	174	43.9
Middle school	168	42.4	Positive	222	56.1
High school	98	24.8	**HER2 status**		
College or higher	70	17.7	Negative	213	53.8
**Family annual income levels**			Positive	183	46.2
Low (T1)	141	35.6	**Ki-67 levels**		
Middle (T2)	128	32.3	<20%	132	33.3
High (T3)	127	32.1	≥20%	264	66.7
**Residence**			**Breast cancer subtypes**		
Urban area	150	37.9	HR+/HER2-negative	163	41.2
Rural area	246	62.1	HR+/HER2-positive	97	24.5
**Family history of breast cancer**			HER2 enriched	86	21.7
No	380	96.0	Triple-negative/basal-like	50	12.6
Yes	16	4.0	**Tumor size stage**		
**BMI levels** (kg/m^2)			1	101	25.5
Underweight (<18.5)	42	10.6	2	230	58.1
Normal weight (18.5-22.9)	245	61.9	3	34	8.6
Overweight (23-24.9)	75	18.9	4	31	7.8
Obese (≥25)	34	8.6	**Node stage**		
**Comorbidity**			0	211	53.3
No	330	83.3	1	104	26.3
Yes	66	16.7	2	61	15.4
**Pre-existing hematological condition**			3	20	5.1
No	280	70.7	**TNM stage**		
Yes	116	29.3	Stage I	76	19.2
**Pre-existing nephrological condition**			Stage II	217	54.8
No	319	80.6	Stage III	84	21.2
Yes	77	19.4	Stage IV	19	4.8
**Pre-existing hepatological condition**			**Histological subtype**		
No	330	83.3	Invasive ductal carcinoma (IDC)	303	76.5
Yes	66	16.7	Non-IDC	43	10.9
			Unknown	50	12.6

Table S2. First-line treatment for breast cancer among study participants.

	All eligible participants (*N* = 396)			All eligible participants (*N* = 396)	
	n	%		n	%
**Breast cancer surgery**			**Taxane types**		
No surgery	22	5.6	Paclitaxel	252	63.6
Modified radical mastectomy	346	87.3	Docetaxel	99	25.0
Radical mastectomy	20	5.1	No taxane	45	11.4
Partial/sub-total mastectomy/ lumpectomy	8	2.0	**Using 5-Fluorouracil**		
			No	313	79.0
**Chemotherapy timing**			Yes	83	21.0
Neoadjuvant	63	15.9	**Dose-dense chemotherapy**		
Adjuvant	333	84.1	No	349	88.1
**Chemotherapy regimens**			Yes	47	11.9
AC-P/FAC-P/ EC-P/FEC-P	216	54.5	**Using G-CSF**		
AC-T/FAC-T/ EC-T/FEC-T	64	16.2	No	290	73.2
AC/EC/ FAC/FEC	40	10.1	Yes	106	26.8
AP/EP/PC/PCBP	34	8.6	**Relative Dose intensity (RDI)[a]**		
AT/ET/TC	33	8.3	RDI≥85%	325	82.1
Monotherapy	9	2.3	RDI<85%	71	17.9
**Sequential anthracycline and taxane**			**Chemotherapy discontinuance**		
No	116	29.3	No	363	91.7
Yes	280	70.7	Yes	33	8.3

[a] RDI: ratio of the dose intensity delivered to the reference standard dose intensity for a chemotherapy regimen; AC: Doxorubicin and cyclophosphamide; EC: Epirubicin and cyclophosphamide; FAC: 5-FU, doxorubicin and cyclophosphamide; FEC: 5-FU, epirubicin and cyclophosphamide; AC-P: AC followed by paclitaxel; FAC-P: FAC followed by paclitaxel; EC-P: EC followed by paclitaxel; FEC-P: FEC followed by paclitaxel; AC-T: AC followed by docetaxel; FAC-T: FAC followed by docetaxel; EC-T: EC followed by docetaxel; FEC-T: FEC followed by docetaxel; AP: Doxorubicin and paclitaxel; EP: epirubicin and paclitaxel; PC: paclitaxel and cyclophosphamide; PCBP: paclitaxel and carboplatin; AT: Doxorubicin and docetaxel; ET: epirubicin and docetaxel; TC: Docetaxel and cyclophosphamide.

Table S3. Association of demographic characteristics and clinical factors with neutropenia.

	Neutropenia (grade ≥3 vs. grade <3)		
	***No.* of grade ≥3/ grade <3**	Model 1 Adjusted OR (95%CI)[1]	Model 2 Adjusted OR (95%CI)[2]
**Age group**			
< 40	19/ 42	1	1
40-49	48/ 105	1.05 (0.55-2.02)	1.24 (0.62-2.50)
50-59	41/ 94	1.07 (0.55-2.09)	1.66 (0.79-3.50)
≥ 60	9/ 38	0.55 (0.22-1.37)	0.82 (0.29-2.32)
**Income levels**			
Low (T1)	37/ 104	1	1
Middle (T2)	38/ 99	1.13 (0.66-1.94)	1.13 (0.63-2.02)
High (T3)	42/ 85	1.26 (0.73-2.16)	1.29 (0.72-2.30)
**Residence**			
Urban area	54/ 96	1	1
Rural area	63/ 183	0.62 (0.40-0.97)	0.53 (0.32-0.87)
**BMI levels**			
Normal weight (18.5-22.9)	72/ 173	1	1
Underweight (<18.5)	11/ 31	0.80 (0.38-1.69)	0.84 (0.38-1.85)
Overweight (23-24.9)	28/ 47	1.43 (0.82-2.49)	1.54 (0.85-2.79)
Obese (≥25)	6/ 28	0.54 (0.21-1.38)	0.55 (0.20-1.50)
**Comorbidity** [a]			
No	103/ 227	1	1
Yes	14/ 52	0.58 (0.29-1.14)	0.56 (0.26-1.17)
**Pre-existing hematological condition** [b]			
No	81/ 199	1	1
Yes	36/ 80	1.15 (0.71-1.86)	1.01 (0.60-1.70)
**Pre-existing nephrological condition** [c]			
No	88/ 231	1	1
Yes	29/ 48	1.63 (0.96-2.77)	1.86 (1.04-3.30)
**Pre-existing hepatological condition** [d]			
No	96/ 234	1	1
Yes	21/ 45	1.19 (0.66-2.14)	1.39 (0.73-2.63)
**TNM stage**			
Stage I	24/ 52	1	1
Stage II	68/ 149	1.01 (0.57-1.80)	0.86 (0.46-1.61)
Stage III-IV	25/ 78	0.76 (0.39-1.50)	0.70 (0.34-1.44)
**Breast cancer subtype**			
HR+/HER2-negative	41/ 122	1	1
HR+/HER2-positive	37/ 60	1.76 (1.01-3.05)	1.69 (0.95-3.01)
HER2 enriched	19/ 67	0.86 (0.46-1.62)	0.71 (0.36-1.39)
Triple-negative/basal-like	20/ 30	2.27 (1.15-4.52)	2.53 (1.22-5.24)
**Sequential anthracycline and taxane**			
No	24/ 92	1	1
Yes	93/ 187	2.00 (1.17-3.42)	1.74 (0.96-3.17)
**Dose-dense chemotherapy**			
No	94/ 255	1	1
Yes	23/ 24	2.73 (1.45-5.15)	2.64 (1.32-5.25)

[1]Multivariable mode 1 was adjusted for age groups at diagnosis, income levels, and residence.; [2] Multivariable model 2 was the multivariable model 1 with additional adjustment for BMI levels, comorbidity, pre-existing hematological, nephrological and hepatological conditions, TNM cancer stage, breast cancer subtype, sequential anthracycline and taxane and dose-dense chemotherapy. ; [a] Having a diagnosis of specific comorbidities, including diabetes mellitus, hypertension, hyperlipidemia, coronary heart disease (CHD), stroke, myocardial infarction, arthritis, lupus, or another chronic disease at enrollment. ; [b] Having at least one of the hematological symptoms (grade≥1), including anemia, neutropenia, lymphopenia, and thrombocytopenia within 120 days prior to chemotherapy. ; [c] Having at least one of the nephrological symptoms (grade≥1), including high creatinine, proteinuria, and hematuria within 120 days prior to chemotherapy. [d] Having at least one of the hepatological symptoms (grade≥1) including high bilirubin, SGOT, and SGPT within 120 days prior to chemotherapy.

Table S4. Association of demographic characteristics and clinical factors with nausea/vomiting.

	Nausea/vomiting (grade ≥3 vs. grade <3)		
	***No.* of grade ≥3/ grade <3**	Model 1 Adjusted OR (95%CI)[1]	Model 2 Adjusted OR (95%CI)[2]
**Age group**			
< 40	10/ 51	1	1
40-49	15/ 38	0.53 (0.22-1.26)	0.50 (0.19-1.30)
50-59	13/ 122	0.52 (0.21-1.29)	0.35 (0.12-1.03)
60+	2/ 45	0.21 (0.04-1.03)	0.09 (0.01-0.61)
**Income levels**			
Low (T1)	16/ 125	1	1
Middle (T2)	14/ 114	0.89 (0.41-1.93)	1.17 (0.48-2.81)
High (T3)	10/ 117	0.59 (0.25-1.38)	0.75 (0.29-1.92)
**Residence**			
Urban area	16/ 134	1	1
Rural area	24/ 222	0.88 (0.44-1.75)	0.98 (0.45-2.10)
**BMI levels**			
Normal weight (18.5-22.9)	33/ 212	1	1
Underweight (<18.5)	3/ 39	0.48 (0.14-1.67)	0.40 (0.11-1.49)
Overweight (23-24.9)	3/ 72	0.29 (0.09-0.98)	0.27 (0.07-0.98)
Obese (≥25)	1/ 33	0.22 (0.03-1.40)	0.15 (0.02-1.33)
**Comorbidity** [a]			
No	32/ 298	1	1
Yes	8/ 58	1.88 (0.76-4.68)	2.91 (1.03-8.24)
**Pre-existing hematological condition** [b]			
No	32/ 248	1	1
Yes	8/ 108	0.58 (0.26-1.32)	0.74 (0.30-1.81)
**Pre-existing nephrological condition** [c]			
No	35/ 284	1	1
Yes	5/ 72	0.54 (0.20-1.44)	0.40 (0.13-1.21)
**Pre-existing hepatological condition** [d]			
No	30/ 300	1	1
Yes	10/ 56	1.84 (0.83-4.09)	2.27 (0.92-5.58)
**TNM stage**			
Stage I	16/ 60	1	1
Stage II	15/ 202	0.25 (0.11-0.55)	0.17 (0.07-0.41)
Stage III-IV	9/ 94	0.32 (0.13-0.81)	0.29 (0.11-0.76)
**Breast cancer subtype**			
HR+/HER2-negative	13/ 150	1	1
HR+/HER2-positive	9/ 88	1.08 (0.44-2.66)	1.23 (0.46-3.27)
HER2 enriched	10/ 76	1.66 (0.68-4.06)	2.56 (0.94-7.00)
Triple-negative/basal-like	8/ 42	2.26 (0.86-5.93)	2.90 (0.98-8.57)
**Sequential anthracycline and taxane**			
No	11/ 105	1	1
Yes	29/ 251	0.94 (0.44-2.00)	1.55 (0.63-3.81)
**Dose-dense chemotherapy**			
No	37/ 312	1	1
Yes	3/ 44	0.59 (0.17-2.02)	0.58 (0.16-2.19)

[1] Multivariable mode 1 was adjusted for age groups at diagnosis, income levels, and residence; [2] Multivariable model 2 was the multivariable model 1 with additional adjustment for BMI levels, comorbidity, pre-existing hematological, nephrological and hepatological conditions, TNM cancer stage, breast cancer subtype, sequential anthracycline and taxane, and dose-dense chemotherapy; [a] Having a diagnosis of specific comorbidities, including diabetes mellitus, hypertension, hyperlipidemia, coronary heart disease (CHD), stroke, myocardial infarction, arthritis, lupus, and another chronic disease at enrollment; [b] Having at least one of the hematological symptoms (grade≥1), including anemia, neutropenia, lymphopenia, and thrombocytopenia within 120 days prior to chemotherapy; [c] Having at least one of the nephrological symptoms (grade≥1), including high creatinine, proteinuria, and hematuria within 120 days prior to chemotherapy; [d] Having at least one the of hepatological symptoms (grade≥1) including high bilirubin, SGOT, and SGPT within 120 days prior to chemotherapy.
